# Linkage Study and Exome Sequencing Identify a *BDP1* Mutation Associated with Hereditary Hearing Loss

**DOI:** 10.1371/journal.pone.0080323

**Published:** 2013-12-02

**Authors:** Giorgia Girotto, Khalid Abdulhadi, Annalisa Buniello, Diego Vozzi, Danilo Licastro, Angela d'Eustacchio, Dragana Vuckovic, Moza Khalifa Alkowari, Karen P. Steel, Ramin Badii, Paolo Gasparini

**Affiliations:** 1 Department of Medical Sciences, University of Trieste, Trieste, Italy; 2 Audiology and Balance Unit, National Program for Early Detection of Hearing Loss, WH, Hamad Medical Corporation (HMC), Doha, Qatar; 3 Wolfson Centre for Age-Related Diseases, King's College London, London, United Kingdom; 4 Institute for Maternal and Child Health- IRCCS “Burlo Garofolo”, Trieste, Italy; 5 CBM S.c.r.l., Trieste, Italy; 6 Molecular Genetics Laboratory, Department of Laboratory of Medicine and Pathology, Hamad Medical Corporation (HMC), Doha, Qatar; Oslo University Hospital, Norway

## Abstract

Nonsyndromic Hereditary Hearing Loss is a common disorder accounting for at least 60% of prelingual deafness. *GJB2* gene mutations, *GJB6* deletion, and the A1555G mitochondrial mutation play a major role worldwide in causing deafness, but there is a high degree of genetic heterogeneity and many genes involved in deafness have not yet been identified. Therefore, there remains a need to search for new causative mutations. In this study, a combined strategy using both linkage analysis and sequencing identified a new mutation causing hearing loss. Linkage analysis identified a region of 40 Mb on chromosome 5q13 (LOD score 3.8) for which exome sequencing data revealed a mutation (c.7873 T>G leading to p.*2625Gluext*11) in the *BDP1* gene (B double prime 1, subunit of RNA polymerase III transcription initiation factor IIIB) in patients from a consanguineous Qatari family of second degree, showing bilateral, post-lingual, sensorineural moderate to severe hearing impairment. The mutation disrupts the termination codon of the transcript resulting in an elongation of 11 residues of the BDP1 protein. This elongation does not contain any known motif and is not conserved across species. Immunohistochemistry studies carried out in the mouse inner ear showed Bdp1 expression within the endothelial cells in the stria vascularis, as well as in mesenchyme-derived cells surrounding the cochlear duct. The identification of the *BDP1* mutation increases our knowledge of the molecular bases of Nonsyndromic Hereditary Hearing Loss and provides new opportunities for the diagnosis and treatment of this disease in the Qatari population.

## Introduction

Hearing loss is the most common sensory deficit in humans. Roughly one child in a thousand is born with hearing impairment significant enough to compromise the development of normal language skills. Hearing loss can be caused by environmental as well as genetic factors or by the combination of both. Hereditary Hearing Loss (HHL) includes a broad range of disorders that affect infants, children and adults [1]. HHL can be conductive (involving the outer ear, the tympanic membrane or the middle ear) and/or sensorineural which involves the inner ear or the acoustic nerve [2]. There are two main forms of HHL, Syndromic (SHHL) (about 15–30% of cases) and Nonsyndromic (NSHHL) (approximately 70%) and they can be transmitted with different patterns of inheritance, the most common being autosomal recessive (approx. 75–80% of all cases). In general, HHL with recessive inheritance shows pre-lingual or post-lingual onset of severe to profound hearing loss with all frequencies affected. In autosomal dominant forms, the phenotype is often less severe, the onset usually post-lingual and the severity ranging from moderate to severe [3]. The pathophysiology reflects the vast genetic and clinical heterogeneity, with many different loci and/or genes associated with auditory dysfunction [4]. According to the HHL homepage, more than 140 NSHHL loci have been mapped, and approximately 65 genes have been identified (see http://hereditaryhearingloss.org/). Based on the type of gene product, these genes can be categorized into several groups such as those coding for proteins involved in the structure and function of hair cells, auditory nerve, and virtually every structural element of the inner ear. As already reported in other studies, HHL especially in Middle Eastern populations is highly heterogeneous, both in the number of genes involved and in the number of alleles at each gene [5]. As regards the Qatari population, a recent study using high-density SNP arrays revealed three clusters consistent with Arabian origin, an eastern or Persian origin and individuals with African admixture [6]. A previous report on HHL in the Qatari population demonstrated a minor role for the *GJB2* gene but no role for *GJB6* or the A1555G mutation, strongly suggesting the presence of additional causative mutations [7]. Here, we report the identification of a gene, never described before as involved in HHL, by linkage study followed by exome sequencing carried out in a NSHHL Qatari family with second degree consanguinity.

## Materials and Methods

### Ethics Statement

Mice. Mouse studies were carried out in accordance with UK Home Office regulations and the UK Animals (Scientific Procedures) Act of 1986 (ASPA) under a UK Home Office licence and the study was approved by the Welcome Trust Sanger Insitute's Ethical Review Committee. Mice were culled using methods approved under this licence to minimize any possibility of suffering.

Human. Consent forms for clinical and genetic studies were signed by each participant and all research was conducted according to the ethical standards as defined by the Helsinki Declaration. The study was approved by the Institutional Review Board of Hamad Medical Corporation (Human subjects ethical compliance document approved 08/06/2009). The research project has been conducted within Qatar (Hamad Medical Corporation) with the strong technical support of the Italian research team that led the data analysis and writing of the manuscript.

### Family Ascertainment and Clinical Diagnosis

**Figure 1:**
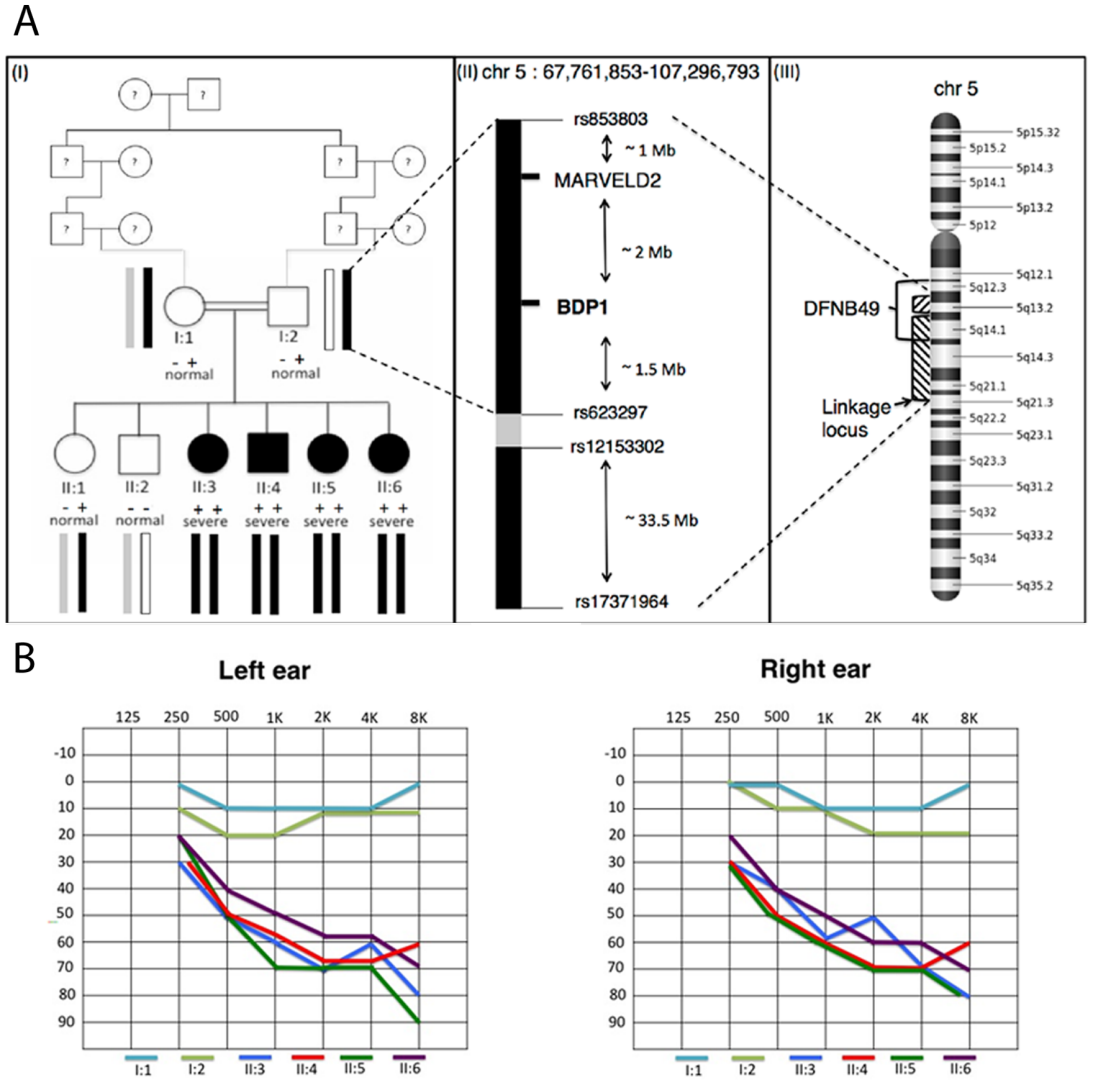
Pedigree and clinical features of the family. **A^I^**, Pedigree of the Qatari family carrying the p.*2625Gluext*11 mutation in *BDP1* gene. Filled symbols represent affected individuals. Double bars indicate the consanguinity of the family. −−: homozygous wild type status, +− heterozygous status, ++ homozygous mutated status. Severe: severe clinical phenotype. Normal: normal hearing function. The haplotypes segregating with the disease and containing a region of 4.5Mb surrounding *BDP1* gene have been shown for each subject. **A^II^**, The markers (rs853803-rs17371964) defining the whole linkage region and the mutual positions of *BDP1* and *MARVELD2* genes are shown in the Figure. Black bars represent the two linkage peaks (LOD score = 3.8) separated by the grey bar with negative LOD score. **A^III^**, The DFNB49 locus (bracket) on chromosome 5 overlapping the linkage region (arrow) is displayed in the Figure. **B**, Audiometric features of the healthy parents (I:1 and I:2) and the patients (II:3, II:4, II:5, II:6) displayed as audiograms (air conduction) and showing the thresholds of the left and right ears. These audiograms represented the latest audiological examination computed in April 2012 when subjects were respectively: I:1 52 years old (y.o.), I:2 53 y.o., II:3 19 y.o., II:4 18 y.o., II:5 16 y.o., II:6 11 y.o. The downward slope indicates that high frequencies were more severely affected than low frequencies, and all patients show a moderate to severe hearing loss. In particular, II:3 is a female patient diagnosed with bilateral moderate to severe NSHHL mainly at High Frequency since she was 4 y.o. II:4 is a male patient diagnosed with bilateral moderate to severe NSHHL since he was 3 y.o. II:5 is a female patient diagnosed with bilateral moderate to severe NSHHL since the girl was 4 y.o. II:6 is a female patient diagnosed with bilateral moderate to severe NSHHL since she was 2 y.o.

A consanguineous family consisting of 8 family members (4 patients, 2 healthy siblings and their healthy parents) was selected for the analysis and included in the study (Figure 1A). Written informed consent was obtained for all study participants after approval from the Unit of Audiology at the Hamad Medical Hospital, Doha, Qatar. The family is characterized by a recessive pattern of inheritance. Affected subjects showed bilateral, sensorineural, early onset, post-lingual, progressive hearing impairment. Pure tone audiometry and otoscopy were performed for all 8 individuals by standard procedures. Individuals II:3, II:4, II:5, II:6 have a moderate to severe form of hearing impairment (Figure 1B). The reported age at which hearing impairment was first noticed was 4 years old (y.o.) for individual II:3, 3 y.o. for individual II:4, 4 y.o. for individual II:5 and 2 y.o. for individual II:6. In particular, a mild hearing loss was initially present involving medium and high frequencies. The progression of the disease led to a gradual involvement of low frequencies and to a moderate-severe clinical phenotype mainly affecting medium and high frequencies. The audiograms in Figure 1B refer to the latest audiological examination of each patient performed in April 2012. At that time the age of subjects was: 52 years old (y.o.) for individual I:1, 53 for individual I:2, 19 y.o. for individual II:3, 18 y.o. for individual II:4, 16 y.o. for individual II:5, 11 y.o. for individual II:6. A clinical and dysmorphological examination of the patients was carried out to exclude non-genetic causes of hearing impairment (for example syphilis, toxoplasmosis, cytomegalovirus, injuries etc.) or the presence of SHHL, but no other relevant feature was detected.

### Genotyping and Linkage Analysis

Genomic DNA was extracted from 300 μl of peripheral whole blood, collected from all family members.

Genotyping was performed using the HumanCytoSNP-12 BeadChip 300K SNPs array. Linkage calculation was performed using the software Merlin for linkage analysis and in particular, a check for Mendelian errors and unlikely recombinations was computed by the Merlin error check, Pedcheck and Pedstats software [8], [9], [10]. Any SNPs found by these error check procedures, were set to missing for all family members. A parametric linkage analysis under a recessive model was performed (using a risk allele frequency 0.00001 and complete penetrance) using Merlin version 1.1.2. The resulting linkage region was then used to filter exome sequence data.

### Exome Sequencing and Data Analysis

Starting from 3 μg of genomic DNA, the exome of five family members (II:3, II:4, II:5, II:6 and I:2) was enriched using SureSelectXT Human All Exon V5 (Agilent Technologies, Inc.) and a whole exome fragment library was then constructed following the manufacturer's protocols (SureSelect Target Enrichment System for the Applied Biosystems SOLiD System-Version 2.0.1). The library quality was assessed using Agilent 2100 Bioanalyzer (Agilent Technologies, Inc.) and then the whole exome library was single-end sequenced (see the manufacturer's protocols, Applied Biosystems SOLiD™ 4 System Templated Bead Preparation Guide and Instrument Operation Guide) on a SOLiD4 platform (Life Technologies).

After the exclusion of all the low quality reads, the 50 bp F3-reads were mapped against the human reference genome (hg19) using Burrows-Wheeler Aligner (BWA) v0.5.9-r16 [11]. All PCR-duplicate reads were removed using Picard-Tools Version: 1.65 (http://picard.sourceforge.net/).

The exome data analysis was limited to the genomic region with a linkage LOD score greater than 3.0. Single nucleotide variants (SNVs) and small insertions and deletions (INDELs) were called by Samtools V0.1.18 [12] and filtered according to a recessive model of inheritance. Low quality variants were filtered out in accordance with the following exclusion criteria: a) sequencing depth coverage less than 20X; or b) quality sequencing score less than 20 (Q20); or c) variation detected on a single DNA strand. All SNVs and INDELs annotations were estimated with ANNOVAR [13] using the RefSeq gene model [14], dbSNP ver137 [15] and the 1000 Genome Project dataset [16].

### Sanger Sequencing

Sanger sequencing was carried out according to standard protocols to confirm SNVs from exome sequencing analysis. Moreover, the whole coding region and 100 nucleotides upstream and downstream of each exon (intron-exon boundaries) of *MARVELD2* gene have been sequenced.

### Expression studies in the Mouse Cochlea

For immunohistochemistry, wild-type mice from the C57BL/6J strain carrying an albino mutation (C57BL/6Brd-Tyr^c-Brd^) [17] at postnatal day five (P5) were culled and dissected in PBS before fixation for two days in 10% formalin at 4°C, washing, dehydrating and embedding in paraffin wax. Embedded samples were cut into 8 µm thick sections along the sagittal plane. Immunohistochemistry was then carried out on slides using the Ventana Discovery machine with the manufacturer's reagents CC1 (cat.no 950–124), EZPrep (cat.no 950–100), LCS (cat.no 650–010), RiboWash (cat.no 760–105), Reaction Buffer (cat.no 95–300), and RiboCC (cat.no 760–107) and according to the manufacturer's instructions. The OmniMap DAB anti-Rb Detection Kit^TM^ (Ventana; cat.no 760–149) with hematoxylin counterstain (cat.no 760–2021) and bluing reagent (cat.no 760–2037) was used. All antibodies were diluted in ‘Antibody staining solution’: 10% fetal calf serum, 0.1% Triton, 2% BSA and 0.5% sodium azide in PBS. The primary antibodies were anti-Bdp1 (Abcam: Ab74415; 1∶50), anti-Lama1 (laminin, alpha 1; Abcam, Cambridge, Uk, cat.no ab11575; 1∶100), anti-Kcnj10 (Alomone Labs, Jerusalem, Israel, cat.no APC-035; 1∶300), while the secondary antibodies used were Epitomics (Burlingame, CA, USA) anti-mouse IgG (3021–1; 1∶500) and Jackson ImmunoResearch (West Grove, PA, USA) biotin conjugated donkey anti-rabbit (711–065–152; 1∶100).

Sections covering the entire inner ear for at least three different mouse samples at P5 were stained, and the observed expression patterns were considered reliable only if present in all three samples. Stained sections were examined and images obtained using an AxioCam HRc camera mounted on a Zeiss microscope. Images were then processed in Photoshop CS5 extended.

## Results

**Figure 2:**
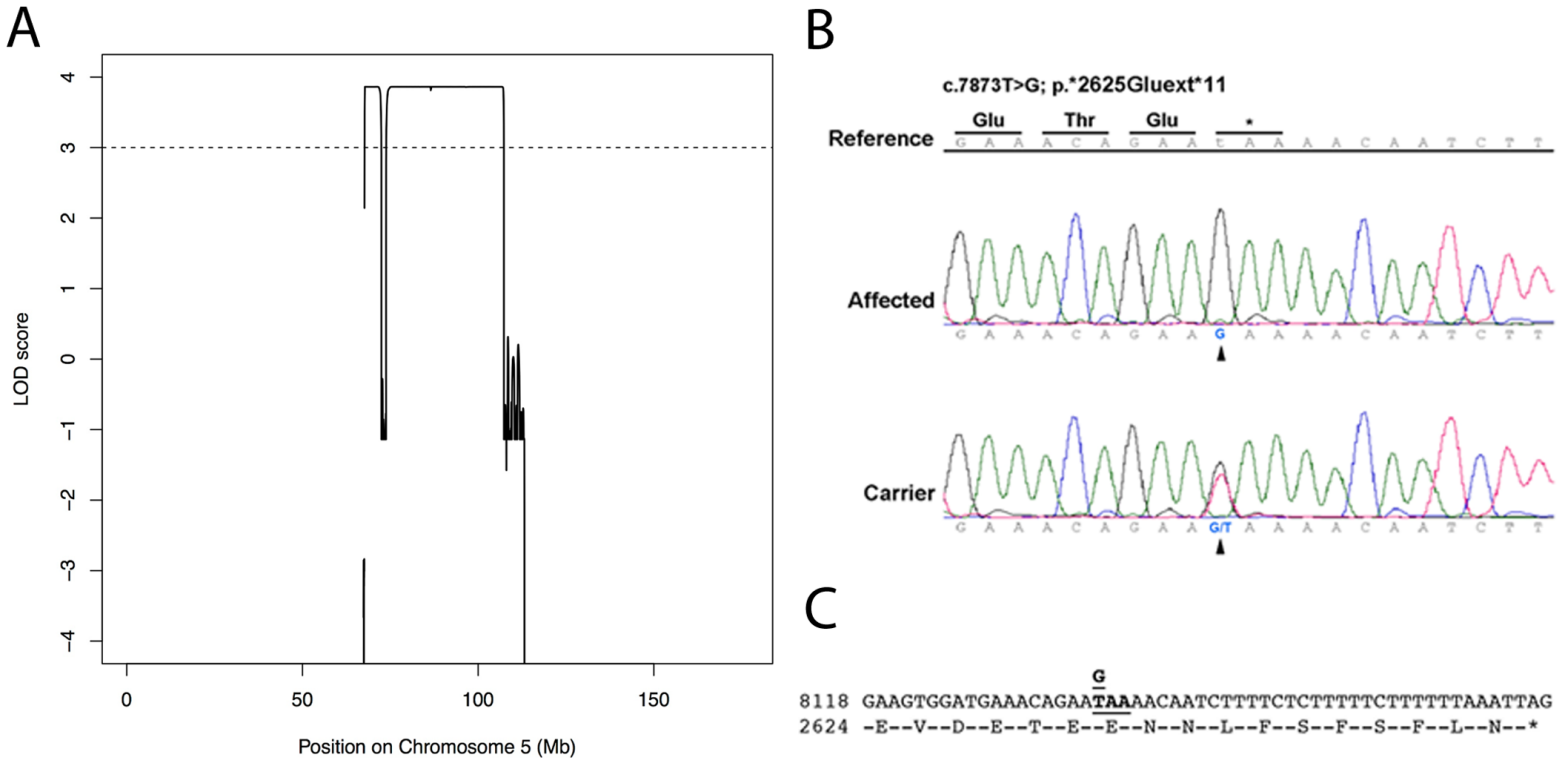
Linkage analysis and exome sequencing results. **A**, identification of a region on chr 5q13 spanning approximately 40 Mb (from 67, 7 Mb (rs853803) to 72,4 Mb (rs623297) and from 73,7 Mb (rs12153302) to 107,3 Mb (rs17371964); and containing a small region (1.3 Mb) with negative LOD score. Chromosome position is reported in the x-axis while LOD score is indicated in the y-axis. **B**, sequence data of the c.7873T>G mutation (p.*2625Gluext*11). *: stop codon disrupted by the mutation. Reference: sequence reference RefSeq NM_018429 Affected: homozygous G/G patient. Carrier: heterozygous G/T subject. **C**, sequence of the *BDP1* gene transcript shows the c.7873 T>G (p.*2625Gluext*11) mutation. This variation changes the termination codon TAA to a coding one GAA (Glu) and introduces a new string of 11 amino acids in the transcript.

Previous molecular studies showed that the family under investigation was negative for the presence of mutations in the most common worldwide NSHHL genes (*GJB2*, *GJB6* and A1555G mitochondrial mutation) [7], suggesting that this family should harbor a mutation in another HHL gene or in a new gene involved in the HL phenotype. Taking into account the power of linkage studies coupled with massively parallel sequencing technologies, we decided to apply these approaches to identify the causative mutation underlying NSHHL in this Qatari family. Linkage analysis revealed a LOD score of 3.8 (corresponding to the estimated maximum achievable LOD score) in a single region spanning approximately 40 Mb on chromosome 5q13 (see Figure 1A and 2A) and containing 270 genes. The region consists of two peaks, the first one ranging from rs853803 to rs623297 (chr5: 67,761,853–72,408,339) and the second one from rs12153302 to rs17371964 (chr5: 73,777,378–107,296,793). All the remaining regions were completely negative in terms of LOD score apart from one with a non-significant LOD score of 1.2 (Table S1 and Figure S1). Within the linkage region, the only gene already known to be involved in NSHHL, *MARVELD2*, was then analysed. Sanger sequence of its coding regions plus intron-exon boundaries (∼100 bp) revealed no pathogenic variants but only a common polymorphism (rs1185246, MAF = 0.477), suggesting that *MARVELD2* was unlikely to be involved in the deafness in this family. Thus, the targeted region identified by linkage analysis (40 Mb) was analyzed by exome sequencing and a total of 8 Gb of sequence data per sample were produced, ensuring an adequate coverage of exons and of approximately 20 nucleotides upstream/downstream of the intron-exon boundaries for each gene. On average 89% of the targeted region had at least 42-fold coverage. After the exclusion of both PCR-duplicates and low quality-reads, an average of 204913 reads were mapped to the targeted region of each sample. Each individual carried on average 195 high-quality genetic variants in this region and, after filtering according to a recessive model of inheritance, 50 exonic SNVs were detected. 44 out of the 50 SNVs have a minor allele frequency (MAF) >2% as reported in dbSNP (NCBI build 137, http://www.ncbi.nlm.nih.gov/projects/SNP/) and therefore were excluded. The remaining 6 SNVs have been further investigated as reported in Table 1. Four of these were excluded because they were synonymous variants. The remaining two genetic variants in *ZNF366* and in *BDP1* genes were respectively a non-synonymous and a stop-loss nucleotide substitution. Only nucleotide variation c.7873 T>G (p.*2625Gluext*11) in the *BDP1* gene was confirmed by Sanger sequencing (Figure 2B), and this mutation segregated with the hearing loss in the whole family (both parents and six siblings, Figure 1A). *BDP1* (B double prime 1, subunit of RNA polymerase III transcription initiation factor IIIB) is located at 5q13.2. The c.7873 T>G (p.*2625Gluext*11) mutation appears to be a deleterious mutation leading to the disruption of the stop codon and introducing the codons for 11 additional amino acids (Figure 2C). Both parents were heterozygous and all the affected offspring were homozygous for the mutation while the healthy siblings were heterozygous (WT/MUT) and homozygous (WT/WT) respectively. This mutation (rs199721728) was present in the heterozygous state with a MAF = 0.0007 in the ESP6500 database (9 out of 8126 alleles of European American ethnicity and 0 out of 3592 of African American), a MAF = 0.0009 in the 1000 Genomes Project database (2/2184 alleles of American ethnicity) and a MAF = 0.004 in dbSNP-v137 (4/1169 of European ethnicity) (http://evs.gs.washington.edu/EVS/, http://www.1000genomes.org/, http://www.ncbi.nlm.nih.gov/SNP/). Moreover, an additional 132 patients' chromosomes from Qatar, 6 from Oman and 52 from Italy were negative for the presence of this variant (our internal database).

**Table 1:** Genetic variants identified in linkage region after removal of SNVs present in dbSNP with a MAF >2%.

Chr	Position	Gene	Ref	Obs	NCBI RS	Variant Category	Nucleotide Substitution	Amino Acids Change	Note
5	71757266	ZNF366	T	C	NM_152625	NS SNV	c.58A>G	p.K20E	NC
5	71757267	ZNF366	C	T	NM_152625	S SNV	c.57G>A	p.V19V	E
5	72192358	TNPO1	A	G	NM_002270	S SNV	c.2217A>G	p.A739A	E
5	75596600	SV2C	G	A	NM_014979	S SNV	c.1683G>A	p.S561S	E
5	98235396	CHD1	A	T	NM_001270	S SNV	c.873T>A	p.T291T	E
5	70860710	BDP1	T	G	NM_018429	stop lost SNV	c.7873T>G	p.*2625Gluext*11	C

Chr: chromosome; Ref: reference base; Obs: observed base; NCBI RS: NCBI Reference Sequence; Variant Category: NS SNV = nonsynonymous single nucleotide variant, S SNV = synonymous single nucleotide variant; Note: NC = Not confirmed by Sanger sequencing, C = Confirmed by Sanger sequencing, E = excluded.

To identify any relevant homology of the extended protein with known motifs/domains, we used the SMART protein database (http://smart.embl-heidelberg.de/). Smart analysis of the full-length protein predicted the presence of a SANT domain (from residue 299 to 347 E score 1.52e-04) and some putative coiled coil and low complexity regions, but nothing related to the eleven amino acid extension sequence. The BLAST protein algorithm (http://blast.ncbi.nlm.nih.gov/Blast.cgi) was also used to search for homologies of the tail, but did not detect any significant hit in Eukaryotes. However, a subset of 5–6 amino acids showed some non-significant homology with proteins of simple organisms such as *Polysphondylium pallidum* etc.. The C-terminal extension contains residues of mixed features: five are bulky hydrophobic residues of which three are the aromatic phenylalanine. The other six comprise four hydrophilic and two acidic amino acids. The overall hydrophobic character of this short stretch containing three aromatic residues suggests that the extension could modify the properties (e.g. fold, aggregation tendency and/or interactions) of the rest of the protein.

**Figure 3:**
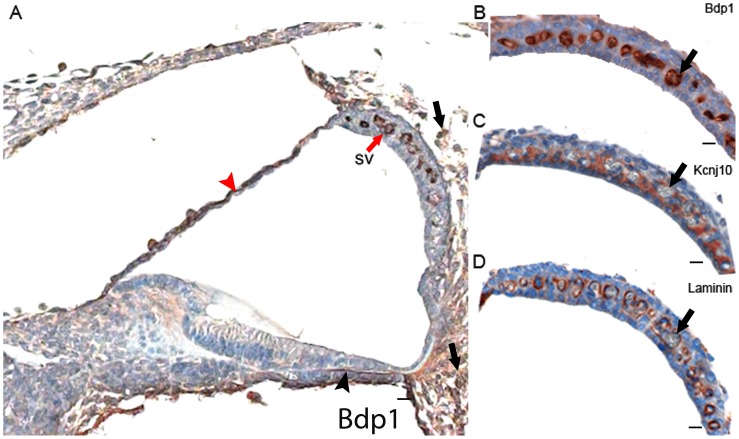
Immunohistochemistry of the mouse cochlea. **A**, Bdp1 protein expression in the cochlea at postnatal day 5. Bdp1 shows expression in the stria vascularis (red arrow), Reissner's membrane (red arrowhead), basilar membrane (black arrowhead), and spiral ligament (black arrow). **B–D**, Bdp1, Kcnj10 and laminin expression in the stria vascularis. Our immunohistochemistry detected Bdp1 protein in the proximity of blood vessels (example of the same blood vessel in adjacent sections is indicated with an arrow in B, C, D). We used Kcnj10 as a marker of intermediate cells (C) and laminin as a marker of the basal lamina surrounding blood vessels (D) in the stria vascularis in the sections shown in A. The expression pattern suggests that Bdp1 is expressed in endothelial cells of the stria vascularis. Scale bars: A: 50 µm; B–D: 10 µm. sv: stria vascularis.

To investigate a possible cochlear role of BDP1, an expression study in the mouse inner ear at postnatal day five was carried out demonstrating a strong pattern of expression in specific cell types in the cochlea. In particular, clear expression is present in the endothelial cells of the stria vascularis capillaries, and in mesenchyme-derived cells and surrounding extracellular matrix around the cochlear duct including the spiral ligament and basilar membrane (Figure 3A). To better understand the localization of this protein in the stria vascularis, we used two different markers: Kcnj10 [18] and laminin [19] (Figure 3C and Figure 3D respectively). Kcnj10 is a membrane channel normally expressed in the strial intermediate cells while laminin marks the basal lamina around strial blood vessels. Comparison of these labelling patterns suggested that Bdp1 was expressed in the endothelial cells (arrows in Figure 3 B–D).

## Discussion

As previously described [7], there is no major gene associated with HHL in the Qatari population. In this report, using a combined approach of linkage studies with massively parallel sequencing, we analyzed a second degree consanguineous family not linked to any known HHL gene. Our results suggested a new gene involved in HHL, named *BDP1*, a member of the *TFIIIB* complex. Transcription factor *TFIIIB* plays key roles in transcription by RNA polymerase III. Its three components (TBP, BRF1 and BDP1) participate in crucial molecular events that include RNA polymerase recruitment, formation of the open initiation complex [20] and recycling of transcription. It has been reported that TFIIIB assembled with certain deletion mutants of its BRF1 and BDP1 subunits is competent in pol III recruitment [21] but the resulting preinitiation complex does not open the promoter. The elongation of the BDP1 protein that we detected could affect the formation of the complex or the opening of the promoter in a similar way, leading to altered transcriptional activity of the protein. BDP1 expression has been reported in many different tissues (see UniGene/ESTProfile database), but in this case the abnormality of the protein apparently affects only the auditory system. We found expression of Bdp1 in specific cell types in the cochlea including endothelial cells of the blood vessels of the stria vascularis, further supporting a role for this gene in auditory function.

Little is known about the molecular architecture of BDP1 except the conservation of its segment of amino acids 299–347 that contains the SANT domain. This domain seems to be the major interaction interface between the TFIIIB components BDP1 and BRF1 and is the most highly conserved sequence in the protein. Apart from this domain, located in the middle of the protein, BDP1 does not show any other specific domains. The predicted mutant BDP1 protein is characterized by an elongation of eleven amino acids, not conserved across species, resulting from a loss of the termination codon. Although the molecular structure of the normal BDP1 protein has not yet been solved we can hypothesize that this extension might influence the structure of the protein possibly leading to a failure to fold correctly as has been described for other abnormal proteins [22]. The *BDP1* gene maps in a hearing loss locus DFNB49, 2Mb away from *MARVELD2*, a gene already known to be involved in hearing loss [23] but containing no predicted pathogenic mutations in this family. All *MARVELD2* mutations so far described are located within the coding region or splice sites, which were included in our sequence analysis. The exclusion of any pathogenic variation in these regions together with the finding of a predicted stop-loss mutation in *BDP1* supports the involvement of the *BDP1* gene mutation in the hearing loss phenotype. Conclusive proof of the involvement of the *BDP1* gene in hearing loss will require the finding of additional mutations in other families associated with deafness or evidence from animal models with Bdp1 mutations, but these are not yet available.

In conclusion, the identification by a combined approach of linkage analysis and exome sequencing of p.*2625Gluext*11 mutation of *BDP1*, a gene in which very few truncating mutations have been described (http://evs.gs.washington.edu/EVS/), its segregation within the large family and the specific cochlear expression pattern of the gene indicate a role for this gene in causing HHL. Moreover, as inherited hearing loss is a highly heterogeneous trait and there are many genes involved that have not yet been identified, the BDP1 gene could be involved in HHL in other populations as well as in the Qatari population. This finding also illustrates the importance of massively parallel sequencing technologies for disease gene identification combined with immunohistochemistry to add tissue-specific expression data. Additional biochemical and functional studies as well as an appropriate mouse model are required to understand better the molecular mechanisms underlying deafness associated with *BDP1* mutations.

## Supporting Information

Figure S1 **Genome wide linkage LOD score.** The genome wide LOD score showing the only significant region on chromosome 5 and containing BDP1 gene is shown. In the x-axis is reported the position of the chromosomes and in the y-axis the LOD score.
(TIFF)

Table S1 **Whole genome results from the linkage analysis.**
(TIFF)

## References

[1] Van Laer L, Cryns K, Smith RJH, Van Camp G (2003) Nonsyndromic hearing loss. Ear and Hearing 24: 275–288. 12923419 10.1097/01.AUD.0000079805.04016.03

[2] Friedman TB, Griffith AJ (2003) Human nonsyndromic sensorineural deafness. Annual Review of Genomics and Human Genetics 4: 341–402. 10.1146/annurev.genom.4.070802.110347 14527306

[3] Van Camp G, Willems PJ, Smith RJ (1997) Nonsyndromic hearing impairment: unparalleled heterogeneity. American Journal of Human Genetics 60: 758–764. 9106521 PMC1712474

[4] Lenz DR, Avraham KB (2011) Hereditary hearing loss: from human mutation to mechanism. Hearing Research 281: 3–10. 21664957 10.1016/j.heares.2011.05.021

[5] Walsh T, Abu Rayan A, Abu Sa'ed J, Shahin H, Shepshelovich J, et al (2006) Genomic analysis of a heterogeneous Mendelian phenotype: multiple novel alleles for inherited hearing loss in the Palestinian population. Human Genomics 2: 203–211. 16460646 10.1186/1479-7364-2-4-203 PMC3525152

[6] Hunter-Zinck H, Musharoff S, Salit J, Al-Ali K a, Chouchane L, et al (2010) Population genetic structure of the people of Qatar. American Journal of Human Genetics 87: 17–25. 20579625 10.1016/j.ajhg.2010.05.018 PMC2896773

[7] Alkowari MK, Girotto G, Abdulhadi K, Dipresa S, Siam R, et al (2011) GJB2 and GJB6 genes and the A1555G mitochondrial mutation are only minor causes of nonsyndromic hearing loss in the Qatari population. International Journal of Audiology 51: 181–5. 22103400 10.3109/14992027.2011.625983

[8] O'Connell JR, Weeks DE (1998) PedCheck: a program for identification of genotype incompatibilities in linkage analysis. American Journal of Human Genetics 63: 259–266. 9634505 10.1086/301904 PMC1377228

[9] Wigginton JE, Abecasis GR (2005) PEDSTATS: descriptive statistics, graphics and quality assessment for gene mapping data. Bioinformatics (Oxford, England) 21: 3445–3447. 10.1093/bioinformatics/bti529 15947021

[10] Abecasis GR, Cherny SS, Cookson WO, Cardon LR (2002) Merlin–rapid analysis of dense genetic maps using sparse gene flow trees. Nature Genetics 30: 97–101. 11731797 10.1038/ng786

[11] Li H, Durbin R (2009) Fast and accurate short read alignment with Burrows-Wheeler transform. Bioinformatics (Oxford, England) 25: 1754–1760. 10.1093/bioinformatics/btp324 PMC2705234 19451168

[12] Li H, Handsaker B, Wysoker A, Fennell T, Ruan J, et al (2009) The Sequence Alignment/Map format and SAMtools. Bioinformatics (Oxford, England) 25: 2078–2079. 10.1093/bioinformatics/btp352 PMC2723002 19505943

[13] Wang K, Li M, Hakonarson H (2010) ANNOVAR: functional annotation of genetic variants from high-throughput sequencing data. Nucleic Acids Research 38: e164. 20601685 10.1093/nar/gkq603 PMC2938201

[14] Pruitt KD, Tatusova T, Klimke W, Maglott DR (2009) NCBI Reference Sequences: current status, policy and new initiatives. Nucleic Acids Research 37: D32–6. 18927115 10.1093/nar/gkn721 PMC2686572

[15] Sherry ST, Ward MH, Kholodov M, Baker J, Phan L, et al (2001) dbSNP: the NCBI database of genetic variation. Nucleic Acids Research 29: 308–311. 11125122 10.1093/nar/29.1.308 PMC29783

[16] 1000 Genomes Project Consortium, Abecasis GR, Altshuler D, Auton A, Brooks LD, et al (2010) A map of human genome variation from population-scale sequencing. Nature 467: 1061–1073. 20981092 10.1038/nature09534 PMC3042601

[17] Linder CC (2001) The influence of genetic background on spontaneous and genetically engineered mouse models of complex diseases. Lab Animal 30: 34–39. 11385732

[18] Wangemann P, Itza EM, Albrecht B, Wu T, Jabba S V, et al (2004) Loss of KCNJ10 protein expression abolishes endocochlear potential and causes deafness in Pendred syndrome mouse model. BMC medicine 2: 30. 15320950 10.1186/1741-7015-2-30 PMC516044

[19] Schnaper HW, Kleinman HK, Grant DS (1993) Role of laminin in endothelial cell recognition and differentiation. Kidney International 43: 20–25. 8433560 10.1038/ki.1993.5

[20] Saïda F (2008) Structural characterization of the interaction between TFIIIB components Bdp1 and Brf1. Biochemistry 47: 13197–13206. 19086269 10.1021/bi801406z

[21] Kassavetis GA, Han S, Naji S, Geiduschek EP (2003) The role of transcription initiation factor IIIB subunits in promoter opening probed by photochemical cross-linking. The Journal of Biological Chemistry 278: 17912–17917. 12637540 10.1074/jbc.M300743200

[22] Zhu Y, Li L, Zhou L, Mei H, Jin K, et al (2011) A novel mutation leading to elongation of the deduced α1(X) chain results in Metaphyseal Chondrodysplasia type Schmid. Clinica Chimica Acta; International Journal of Clinical Chemistry 412: 1266–1269. 21447328 10.1016/j.cca.2011.03.026

[23] Riazuddin S, Ahmed ZM, Fanning AS, Lagziel A, Kitajiri S, et al (2006) Tricellulin is a tight-junction protein necessary for hearing. American Journal of Human Genetics 79: 1040–1051. 17186462 10.1086/510022 PMC1698716

