# Supplementary figures and images for: Linkage Study and Exome Sequencing Identify a BDP1 Mutation Associated with Hereditary Hearing Loss
Source: PLoS One. 2013 Dec 2;8(12):e80323. doi: 10.1371/journal.pone.0080323 (PMC3846559; doi:10.1371/journal.pone.0080323)

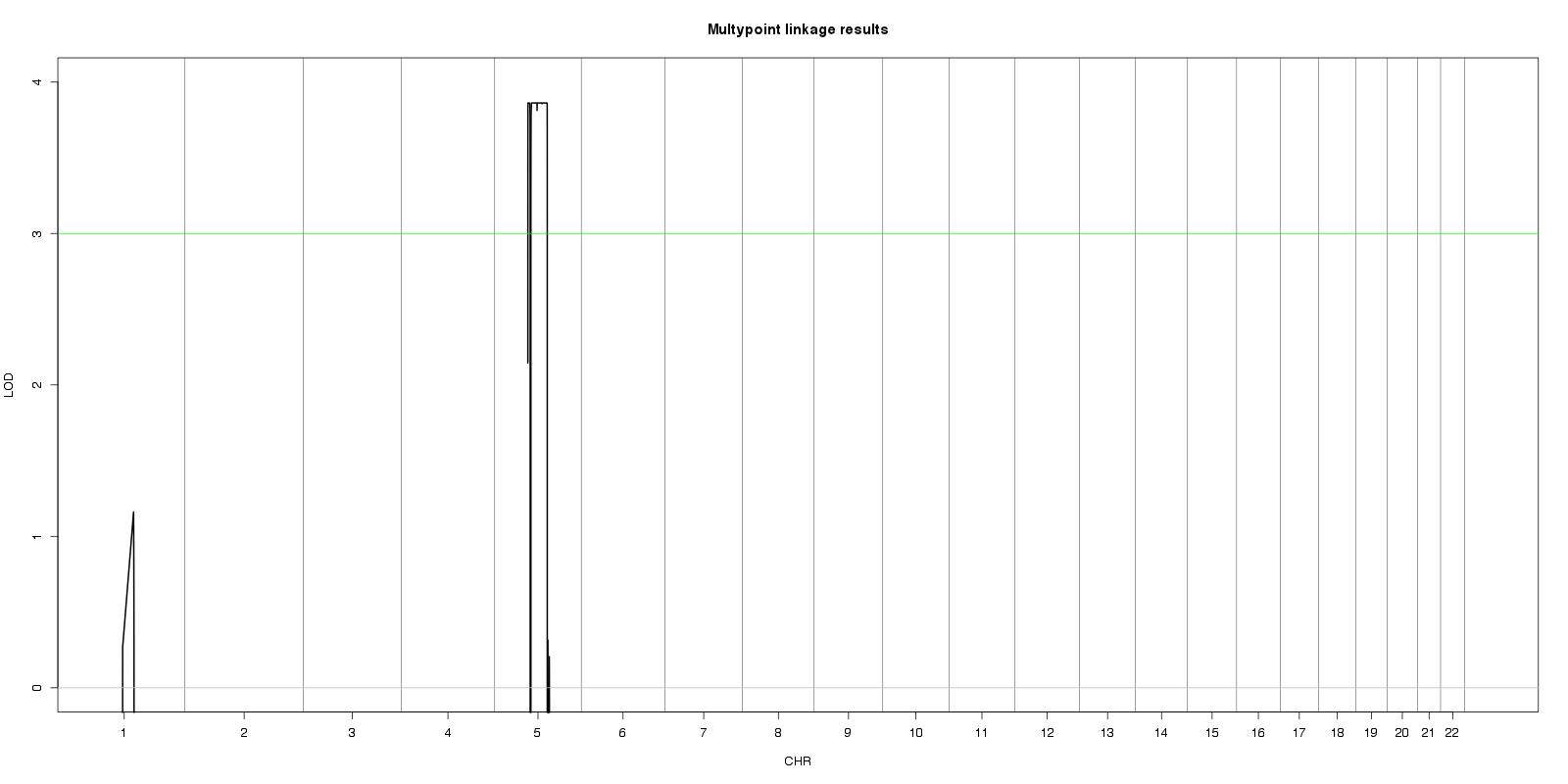

Supplement: Figure S1 — Genome wide linkage LOD score. The genome wide LOD score showing the only significant region on chromosome 5 and containing BDP1 gene is shown. In the x-axis is reported the position of the chromosomes and in the y-axis the LOD score. (TIFF) [file pone.0080323.s001.tiff]

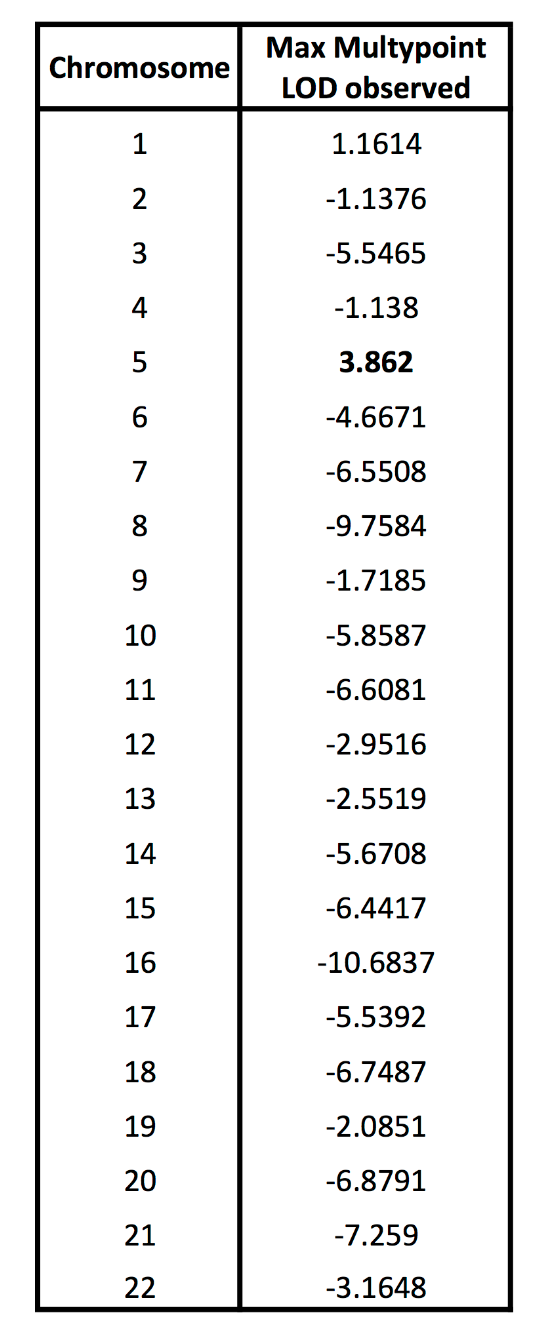

Supplement: Table S1 — Whole genome results from the linkage analysis. (TIFF) [file pone.0080323.s002.tiff]
